# Nursing students’ access to technological devices in nursing education: A quantitative study in Namibia

**DOI:** 10.4102/curationis.v48i1.2626

**Published:** 2025-02-21

**Authors:** Emma M. Nghitanwa, Erkkie Haipinge, Lukas M. Josua

**Affiliations:** 1 School of Nursing and Public Health, Faculty of Health Sciences and Veterinary Medicine, University of Namibia, Windhoek, Namibia; 2 Centre for Innovation in Learning and Teaching, University of Namibia, Windhoek, Namibia; 3 Department of Higher Education and Lifelong Learning, University of Namibia, Windhoek, Namibia

**Keywords:** integration, higher education, nursing education, student nurse, technological devices

## Abstract

**Background:**

Technology integration in higher education is a critical aspect of the 21st century, as it enhances student learning.

**Objectives:**

This study aimed to assess students’ access to and utilisation of technological devices, as well as the integration of technology in nursing education at a higher education institution.

**Method:**

A quantitative approach with a descriptive, cross-sectional study design was used. The study was conducted at a public university in Windhoek. Data were collected via a self-administered questionnaire among 70 third-year nursing students pursuing a bachelor’s degree. The data were analysed using SPSS version 26.

**Results:**

Majority (74.2%) of the participants were aged 20–25 years, 82.9% were female, and 98.6% were single. More than half (58.6%) were Oshiwambo-speaking. Most participants (47.1%) owned technological devices. The majority (60.0%) preferred laptop computers, and the majority used cell phones, messaging apps, social media and WhatsApp daily for learning and communication. Most participants used technological devices to download learning materials, submit online assessments and contact lecturers and classmates. Most preferred a blended mode of learning, online formative assessments and face-to-face examinations.

**Conclusion:**

The study found that most students owned and utilised technological devices, which facilitated blended learning during the coronavirus disease 2019 (COVID-19) pandemic. Technology integration improved access to learning, as students were able to attend classes from locations with Internet access.

**Contribution:**

The study contributes to the body of knowledge on technology integration in higher education and highlights the importance of transforming higher education through the effective use of technology.

## Introduction

Technology integration in higher education benefits both students and lecturers, particularly in nursing education. It is defined as the intentional use of technology to promote learning, increase productivity and enhance performance (Mdhlalose & Mlambo 2023). However, before integrating technology into nursing education, it is essential to conduct a needs assessment to evaluate students’ digital literacy and the availability of devices for online learning. Technologies such as web-based platforms, mobile devices, computers, tablets, internet applications and multimedia classrooms play a crucial role in improving the quality of education (Karkouti 2023). To effectively implement technology in a learner-centred environment, educators must possess appropriate pedagogical, technological and content-based knowledge, and they should understand the interaction between these domains (Hsiao-Jung et al. 2019). In addition, access to technological devices is vital for the successful delivery of teaching and learning.

### Background

The World Health Organization (WHO) declared coronavirus disease 2019 (COVID-19) a global pandemic on 30 January 2020 (Liguori & Winkler 2020). In response, many higher education institutions (HEIs) worldwide transitioned to blended or fully online learning. At the University of Namibia (UNAM), Emergency Remote Teaching (ERT) was implemented, which required a blended learning model combining face-to-face and remote instruction to continue education while minimising the risk of COVID-19 transmission among students and staff. For effective remote learning, students must have access to technological devices, and these devices must be integrated into the teaching process by both students and lecturers. Technological devices, such as smartphones, laptops and desktops, are essential in higher education. The availability of these devices, their ease of use and the interest they generate were found to enhance learning, compared to traditional teaching methods (Mdhlalose & Mlambo 2023). Technology facilitates teaching by enabling lecturers to employ various methods that cater to diverse learning needs. For example, students can listen to audio materials or watch videos multiple times to reinforce their understanding. In addition, cognitive skills and creativity are enhanced by incorporating virtual applications into the curriculum (Steele et al. 2019). Technology integration increases student interest and participation, optimising learning outcomes (Haleem et al. 2022). Smyth et al. (2011) argue that nursing and midwifery students’ learning is enhanced through blended learning approaches. The shift to blended learning at HEIs in Namibia was largely driven by the COVID-19 pandemic. However, no study has been identified that specifically examines nursing students’ access to technological devices. This study aims to assess nursing students’ access to technological devices to support technology integration in nursing education

### Aim

The study aimed to assess students’ access to and utilisation of technological devices, as well as the integration of technology in nursing education at an HEI.

### Objectives

The objectives of the study were to:

- Determine students’ access to technological devices for learning and teaching.
- Identify the preferred technological devices for accessing the Internet for learning and teaching.
- Assess students’ use of technological devices.
- Identify the common technology modes used in teaching and learning.
- Determine students’ preferred learning and assessment methods.

## Research methods and design

### Study design

A quantitative, descriptive, cross-sectional study design was used.

### Study setting

The study was conducted at a public university in Windhoek, the capital city of Namibia.

### Population and sampling

The study population consisted of 85 third-year nursing students. Census sampling was employed to include all students in the study, given the small population size and the absence of prior studies at the specific university. A total of 70 students agreed to participate. The inclusion criteria involved all third-year bachelor’s degree nursing students on the selected campus. The class register was obtained from the lecturer and used as the sampling frame. Participants were recruited directly by the researcher, who approached them on campus during their theoretical placement. An information sheet explaining the study’s aims and objectives was provided, along with consent forms for interested individuals to sign, indicating their willingness to participate. Participants who consented were given a questionnaire to complete.

### Data collection and management

Data were collected in September 2020 using a self-administered questionnaire developed in English by the researcher. All participants were proficient in English, which is the official language and the medium of instruction in higher education in Namibia. The questionnaire consisted of four sections: Section one covered sociodemographic characteristics. Section two focussed on students’ access to and utilisation of technological devices. Section three addressed preferred modes of teaching and learning. Section four gathered information on preferred learning and assessment methods. Participants were given four weeks to complete the questionnaires, which were then deposited in a secure, locked box. The box was stored in the administrator’s office for security, and only the researcher held the key. The researcher collected the box after the data collection period.

### Data analysis

Data were entered and analysed using the Statistical Package for the Social Sciences (SPSS) version 26.0 (IBM Corp., Armonk, New York, United States). Descriptive univariate analysis was performed for each variable, producing frequencies and percentages.

### Validity and reliability

To ensure content validity; the data collection tool was reviewed by a mentor who is an education expert, to assess the clarity, relevance and simplicity of the content. Construct validity was ensured by developing the tool based on existing knowledge. A Cronbach’s alpha was calculated to ensure internal consistency, yielding a value of 0.829. Reliability was further ensured by piloting the data collection tool on 10% of the study population to confirm that all relevant variables were covered. Changes were made to some questions which were not clear to participants following the pilot study, and the participants involved in the pilot were excluded from the main study.

### Ethical considerations

Ethical approval to conduct this study was obtained from the UNAM School of Nursing Research Ethics Committee (reference number: SoNEC/2020). Participants were recruited from third-year bachelor’s degree nursing students at the selected institution. They were informed of the voluntary nature of participation and the study’s aims were explained. Informed consent was obtained after participants read the information sheet. Anonymity was ensured by instructing participants not to write their names on the questionnaire, preventing identification or linking responses to individuals. Justice was maintained by selecting participants fairly based on the inclusion criteria, without discrimination. Non-maleficence was ensured by avoiding questions that could cause emotional distress.

## Results

### Sociodemographic characteristics of the participants

As shown in Table 1, the majority of participants (*n* = 52; 74.2%) were in the age category of 20–25 years. Most participants (*n* = 58; 82.9%) were female and a large proportion (*n* = 41; 58.6%) were Oshiwambo-speaking. In addition, the vast majority (*n* = 67; 98%) were single.

**TABLE 1:** Sociodemographic characteristics of the participants.

Variable	*n*	%
**Age category (years)**		
20–25	52	74.2
26–30	9	13.0
31–35	8	11.4
36–40	1	1.4
**Gender**		
Male	11	15.7
Female	58	82.9
Do not want to state	1	1.4
**Marital status**		
Single	69	98.6
Married	1	1.4
**Language**		
Oshiwambo	41	58.6
English	8	11.4
Afrikaans	3	4.3
Rukwangali	3	4.3
Other language	15	21.4

### Access to technological devices

**FIGURE 1:**
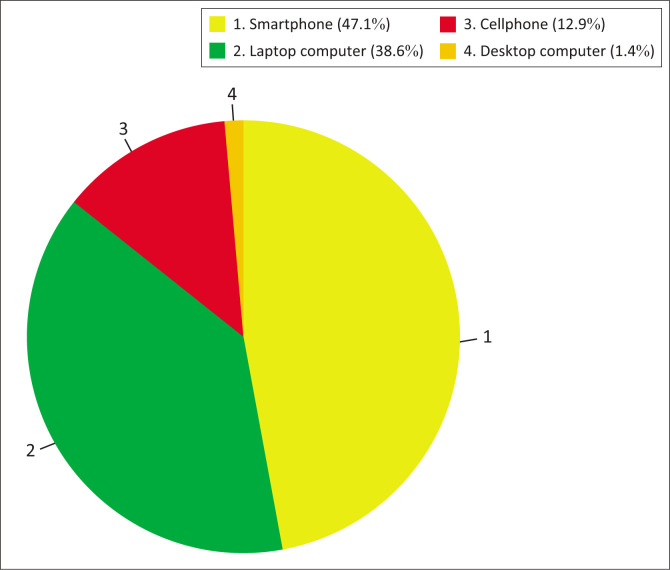
Access to technological devices: Percentage share of different devices owned by participants.

Participants were asked to indicate the types of technological devices they owned, and the results are presented in Figure 1. Nearly half (*n* = 33; 47.1%) reported owning smartphones with Internet access, while a few (*n* = 9; 12.9%) owned cell phones that could not connect to the Internet.

### Preferred technological devices to access the Internet

**FIGURE 2:**
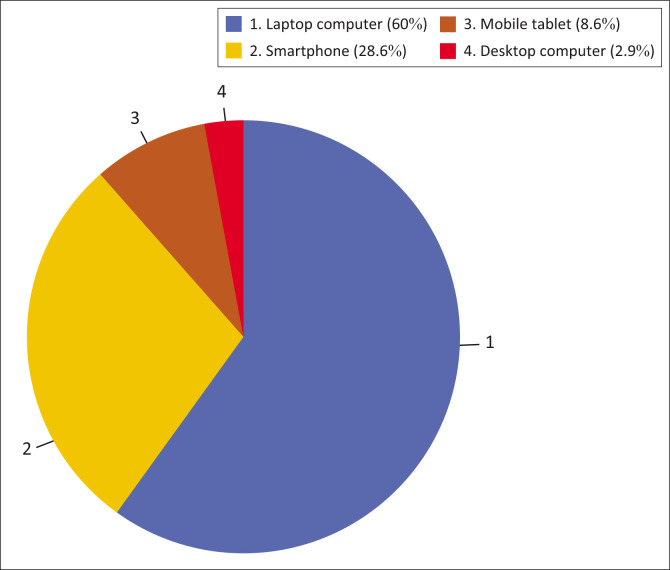
Technological devices preferred to access the Internet: Percentage share of different devices owned by participants.

Regarding preferences for digital devices, most participants (*n* = 42; 60.0%) preferred laptops, while only a small number (*n* = 2; 2.8%) preferred desktop computers, as shown in Figure 2.

### Technological devices usage

Participants were also asked to indicate the frequency of their technological device usage, and the results are displayed in Table 2. Most participants (*n* = 45; 64.3%) reported making cell phone calls daily, while only a small proportion (*n* = 2; 2.9%) made calls less than monthly. Furthermore, the majority (*n* = 65; 92.9%) sent text messages daily, while a few (*n* = 5; 7.1%) sent text messages weekly. Regarding the frequency of paying bills or conducting online banking, the majority (*n* = 36; 51.4%) reported doing so monthly, while some (*n* = 14; 20.0%) indicated that they had never used online banking. On the question of how often participants downloaded information online, nearly half (*n* = 32; 45.7%) did so weekly, and a smaller group (*n* = 3; 4.3%) reported downloading information less than monthly. When asked about uploading information online, a significant number of participants (*n* = 21; 30.0%) did so weekly, while a smaller group (*n* = 16; 22.9%) uploaded information daily, and a few (*n* = 4; 5.8%) stated they had never uploaded information online. In terms of social media usage, the majority (*n* = 42; 60.0%) used social media daily, while a small minority (*n* = 1; 1.4%) reported never using social media. Regarding WhatsApp usage, the majority (*n* = 59; 84.3%) used it daily, while a few (*n* = 2; 2.9%) used it monthly or less than monthly. In addition, many participants (*n* = 17; 24.3%) reported never streaming music, while a similar number (*n* = 17; 24.3%) indicated that they played games on an iPad, phone or similar device weekly. Finally, when asked about reading e-books, a notable portion of participants (*n* = 24; 34.3%) did so monthly, while a smaller group (*n* = 14; 20.0%) reported reading e-books weekly and some (*n* = 10; 14.3%) stated they had never read an e-book.

**TABLE 2:** Technological device usage.

Variable	Daily		Weekly		Monthly		Less than monthly		Never	
	*n*	%	*n*	%	*n*	%	*n*	%	*n*	%
Make a cell phone call	45	64.3	17	24.3	6	8.6	2	2.9	0	0.0
Send a text message	65	92.9	5	7.1	0	0.0	0	0.0	0	0.0
Bank or pay bills online	1	1.4	3	4.3	36	51.4	16	22.9	14	20.0
Download information from online	25	35.7	32	45.7	10	14.3	3	4.3	0	0.0
Upload information online	16	22.9	21	30.0	15	21.4	14	20.0	4	5.7
Use social media (Facebook, Twitter)	42	60.0	14	20.0	8	11.4	5	7.2	1	1.4
Use messaging application (WhatsApp)	59	84.2	7	10.0	2	2.9	2	2.9	0	0.0
Stream music	13	18.6	13	18.6	13	18.6	14	20.0	17	24.2
Play games on an iPad, phone or equivalent device	16	22.9	17	24.2	12	17.1	9	12.9	16	22.9
Read e-books	9	12.9	14	20.0	24	34.3	6	8.6	17	24.2

### Common technology modes used in teaching and learning

The results for the technology modes used in teaching and learning are shown in Table 3. A significant number of participants (*n* = 30; 42.9%) indicated that they ‘almost always’ use websites to learn more about their courses. Similarly, nearly half (*n* = 34; 48.6%) reported that they ‘almost always’ use word processing software to write assignments, and more than half (*n* = 41; 58.6%) stated that they ‘almost always’ download learning materials. Most participants (*n* = 48; 68.6%) reported ‘almost always’ submitting assignments using a computer or another digital device. In terms of communication with lecturers via email, WhatsApp, or course forums, the majority (*n* = 56; 80.0%) indicated that they ‘almost always’ use these methods. For communication with fellow students via email, WhatsApp, or course forums, half of the participants (*n* = 35; 50.0%) stated they ‘almost always’ do so. Regarding online meetings with lecturers, a significant number (*n* = 22; 31.7%) reported doing this ‘often’. As for contacting lecturers via WhatsApp, nearly half (*n* = 31; 44.3%) indicated that they ‘almost always’ use this method. When it came to contacting lecturers via phone calls, most participants (*n* = 26; 37.1%) reported doing so ‘sometimes’, while a small minority (*n* = 4; 5.7%) indicated that they ‘never’ contacted lecturers by phone. For contacting lecturers via SMS, a notable number (*n* = 20; 28.6%) reported doing this ‘sometimes’. In terms of communication via Moodle discussion forums, nearly half of the participants (*n* = 33; 47.1%) indicated that they used this platform ‘often’ to communicate with lecturers, while a notable number (*n* = 26; 37.1%) reported using virtual classrooms ‘often’ for communication. When asked about using email to contact classmates, most participants (*n* = 25; 35.7%) indicated doing so ‘sometimes’. Regarding chatrooms, a significant number (*n* = 20; 28.1%) of participants indicated using them ‘sometimes’ to communicate with classmates. In contrast, a large proportion (*n* = 48; 68.6%) reported ‘almost always’ using text messages to contact classmates. The majority (*n* = 37; 52.9%) of participants also reported ‘almost always’ using phone calls to communicate with classmates. For communication via Moodle discussion forums, a notable number (*n* = 26; 37.1%) of participants indicated doing so ‘often’. In terms of in-person communication with classmates, a significant number (*n* = 31; 44.3%) reported meeting ‘often’. Lastly, when asked about virtual meetings with classmates, a notable portion (*n* = 30; 43.3%) reported meeting virtually ‘sometimes’.

**TABLE 3:** Technology modes used in teaching and learning.

Variable	Almost always		Often		Sometimes		Rarely		Never	
	*n*	%	*n*	%	*n*	%	*n*	%	*n*	%
I use websites that help me learn more about my courses	30	42.9	22	31.4	10	14.3	8	11.4	0	0.0
I use word processing to write assignments	35	50.0	17	24.3	14	20.0	3	4.3	1	1.4
I download learning materials	41	58.6	16	22.9	12	17.1	0	0.0	1	1.4
I submit assignments using a computer or another digital device	48	68.6	17	24.3	4	5.7	1	1.4	0	0.0
Frequencies of using emails, WhatsApp apps, or course forums when communicating with lecturers	56	80.0	9	13.0	3	4.0	2	3.0	0	0.0
Frequency of using emails, WhatsApp apps, or course forums when communicating with fellow students	35	50.0	21	30.0	12	17.0	2	3.0	0	0.0
Frequency of meeting online with the lecturers	17	24.2	22	31.4	17	24.3	8	11.4	6	8.7
I use WhatsApp to contact a lecturer	31	44.3	24	34.2	10	14.3	4	5.8	1	1.4
I contact the lecturers via phone calls	12	17.1	18	25.7	26	37.1	10	14.3	4	5.8
I use SMS messages to contact a lecturer	18	25.7	17	24.3	20	28.6	9	12.8	6	8.6
I communicate with lecturers on the Moodle discussion forum	24	34.3	33	47.1	11	15.7	2	2.9	0	0.0
I communicate with lecturers using a virtual classroom	13	18.6	26	37.1	17	24.3	8	11.4	6	8.6
I use emails to contact my classmates	7	10.0	7	10.0	25	35.7	17	24.3	14	20.0
I contact my classmates via chatrooms	13	18.6	19	27.2	20	28.6	8	11.4	10	14.2
I contact my classmates using text messages	48	68.7	15	21.4	5	7.1	1	1.4	1	1.4
I use phone calls to communicate with my classmates	37	52.9	14	20.0	15	21.3	2	2.9	2	2.9
I communicate with my classmates using Moodle Discussion	24	34.3	21	30.0	19	27.1	1	1.4	5	7.2
I communicate with my classmates by meeting in person	17	24.3	31	44.3	13	18.6	3	4.2	6	8.6
I met with my classmates virtually	10	14.2	15	21.4	30	43.0	3	4.3	12	17.1

SMS, short message service.

### Preferred learning and assessment methods

The results regarding participants’ preferred learning and assessment methods are displayed in Table 4. In terms of preferred course delivery modality, the majority (*n* = 41; 58.6%) preferred a combination of face-to-face and online teaching (blended learning), while a significant number (*n* = 19; 27.1%) preferred face-to-face instruction in a traditional classroom setting, and a small group (*n* = 10; 14.3%) favoured fully online teaching. Regarding preferences for taking tests, most participants (*n* = 42; 60.0%) preferred online assessments, while smaller groups, namely, *n* = 16 (22.9%) and *n* = 12 (17.1%) participants preferred blended mode of assessments and face-to-face assessments, respectively. In terms of examinations, the majority (*n* = 55; 78.6%) indicated a preference for face-to-face examinations, with a smaller group (*n* = 5; 7.1%) preferring a blended mode of examination. Concerning assignment submission, the majority (*n* = 52; 74.3%) preferred submitting assignments online. Although most students preferred online formative assessments, the majority (*n* = 55; 78.6%) favoured face-to-face summative assessments.

**TABLE 4:** Preferred learning and assessment methods.

Variable	Face to face		Online		Blended learning mode	
	*n*	%	*n*	%	*n*	%
The study mode preferred to take the course	19	27.1	10	14.3	41	58.6
The preferred mode for taking tests	12	17.1	42	60.0	16	22.9
The preferred mode for assignment submission	17	24.3	52	74.3	1	1.4
Preferred mode to take an examination	55	78.6	10	14.3	5	7.1

## Discussion

The findings of this study indicate that the majority of participants were middle-aged and female. This age group may be more technologically inclined compared to the older age group (36–40 years), which was underrepresented in the study. Teresa-Morales et al. (2022) have noted that nursing is traditionally a female-dominated profession, which may explain the high percentage of female participants in this study. In addition, the study reveals that most participants were Oshiwambo-speaking. According to the Namibia 2023 Population Census report, the Wambo ethnic group constitutes the majority in Namibia (Namibia Statistics Agency 2024). The current findings also suggest that most participants had access to technological devices, highlighting the advantages of accessibility in technological integration

The study reveals that the majority of participants preferred using laptop computers for learning and teaching. It also highlights the extensive use of technological devices for activities such as making calls, paying bills, downloading and uploading information. The integration of technology – such as web-based technologies, mobile devices and applications, computers, tablets and multimedia – is essential for enhancing the quality of education. Furthermore, virtual learning and technology integration in higher education increase accessibility for any student with Internet access (Mdhlalose & Mlambo 2023). To effectively support learner-centred education, educators need appropriate pedagogical, technological and content-based knowledge, as well as an understanding of the interaction between these domains (Hsiao-Jung et al. 2019). The availability of equipment, ease of use and interest in integrating technology into classrooms – compared to traditional teaching methods – have been shown to improve student learning and enhance digital literacy (Porter & Graham 2016). Most participants in this study reported using mobile phones, consistent with other literature, which suggests that mobile phones are the most prevalent information communication technology (ICT) tools in the developing world, with a rapidly increasing penetration rate (Rotondi et al. 2020). Participants also indicated daily use of technology to communicate with lecturers and fellow students. These findings support the claims of Olapiriyakul and Scher (2006), who argued that the goal of technology infrastructure is to support instructional technology, enhance communication between students and instructors, and create learning communities. This study confirms that students create online communities, such as WhatsApp groups, to facilitate communication with their peers and lecturers. These online communities promote knowledge sharing, enable students to exchange expertise and values, and encourage collaboration through multi-user software and course tutorials (Georgina 2007). Damewood (2016) emphasised the need for educational institutions to ensure adequate repair and support personnel to address the increased maintenance demands resulting from the widespread use of technology. Most participants in this study preferred blended learning. Janes et al. (2023) reported several advantages of blended learning in nursing education, such as successfully achieving learning objectives, increased student-student and student-lecturer interaction, greater flexibility and enhanced learning outcomes compared to traditional courses. The learning process is further improved by combining face-to-face teaching with technology integration, allowing students to access open online resources, including educational websites. Virtual applications integrated into the curriculum can enhance cognitive and creative skills, fostering a student-centred learning environment (Steele et al. 2019). However, some students expressed a preference for traditional face-to-face learning, which may stem from a lack of training in information technology. To address this, the university has incorporated a computer studies module for all undergraduate courses to improve information technology (IT) literacy. Spotts (1999) recommended providing technological support and academic recognition to lecturers, along with adequate time and training, to ensure the successful integration of technology in education.

The integration of technology in teaching and learning enables remote education. For example, during the COVID-19 pandemic, when meetings were restricted to small groups, technology allowed lecturers to continue using platforms such as Big-Blue Button, Microsoft Teams, Moodle Learning Management System, WhatsApp and email. Technology integration improved access to education by allowing students to attend classes from anywhere, provided they had technological devices and Internet access. During COVID-19 lockdowns, students could participate in online classes without changing their geographical location (Butler et al. 2016). This led to cost savings, as students did not need to pay for accommodation or transportation to campus. Damewood (2016) further argued that the increased use of technology requires educational institutions to have sufficient personnel to maintain and repair equipment. The study also found that most students were able to read e-books, use websites for learning and submit assessments online. Pérez-Marín, Santacruz and Gómez (2012) proposed a methodology for blended learning that serves as a repository for digital course materials, provides an additional communication channel between lecturers and students, and allows for the collection and evaluation of student work. In addition, online assessment systems offer immediate feedback to students and provide more opportunities for formative assessment. Sáiz-Manzanares, Escolar-Llamazares and Arnaiz González (2020) also noted that blended learning allows for virtual class discussions using tools such as virtual microphones, which promote active learning. Lecturers can also take attendance online to monitor student participation. Technological integration increases the likelihood of students meeting course outcomes compared to fully online or fully face-to-face courses by reducing dropout rates, improving test scores and boosting student motivation (Qamar et al. 2024). The findings of this study suggest that students are embracing the integration of technology in nursing and midwifery education.

### Limitation

The study was conducted on only one campus of the HEI; therefore, the findings may not be generalisable to other campuses or institutions.

### Recommendations

Given the increasing acceptance and integration of technology in nursing education, HEIs should ensure adequate student support, including the provision of Internet data for connectivity and training in digital technologies. Lecturers are encouraged to actively participate in technology integration, and institutions should provide periodic training and support for both lecturers and students in the effective use of technology.

## Conclusion

The study highlights the accessibility of technological devices among the majority of students, which facilitated the integration of technology in teaching and learning in higher education. The findings indicate that participants were actively using technological devices for learning and teaching. Technology access and utilisation played a key role in successfully facilitating blended learning during the COVID-19 pandemic. In addition, blended learning emerged as the preferred method of teaching and learning, as it enhances the integration of technology in education.
